# Delirium and Cognitive Screening in National Hip Fracture Registries: Scoping Review Protocol

**DOI:** 10.12688/hrbopenres.13996.2

**Published:** 2025-01-24

**Authors:** Niamh A. Merriman, Rose S. Penfold, Louise Brent, Pamela Hickey, Mary E. Walsh, Eithne Sexton, Tara Coughlan, Alasdair M. J. MacLullich, Antony Johansen, Cristina Ojeda-Thies, Andrew J. Hall, Catherine Blake

**Affiliations:** 1 School of Public Health, Physiotherapy and Sports Science, University College Dublin, Dublin, Ireland; 2 Edinburgh Delirium Research Group, Ageing and Health and Advanced Care Research Centre, University of Edinburgh, Edinburgh, Scotland, UK; 3 National Office of Clinical Audit, Dublin, Ireland; 4 School of Pharmacy and Biomolecular Science, Royal College of Surgeons in Ireland University of Medicine and Health Sciences, Dublin, Ireland; 5 School of Population Health, Royal College of Surgeons in Ireland University of Medicine and Health Sciences, Dublin, Ireland; 6 Discipline of Medical Gerontology, School of Medicine, Trinity College Dublin, Dublin, Ireland; 7 Department of Age-related Healthcare, Tallaght University Hospital, Dublin, Ireland; 8 School of Medicine, Cardiff University and University Hospital of Wales, Cardiff, Wales, UK; 9 National Hip Fracture Database (NHFD), Falls and Fragility Fracture Audit Programme, Royal College of Physicians, London, England, UK; 10 Department of Surgery, School of Medicine, Complutense University of Madrid, Madrid, Spain; 11 Department of Traumatology and Orthopaedic Surgery, Hospital Universitario 12 de Octubre, Madrid, Spain; 12 School of Medicine, University of St Andrews, St Andrews, Scotland, UK

**Keywords:** scoping review protocol, hip fracture, audit, registries, cognition, delirium, older adults

## Abstract

**Background:**

Delirium and cognitive impairment are common in hip fracture populations and are associated with significant adverse patient outcomes. National hip fracture registries facilitate improvements in patient outcomes and care quality, such as reduced mortality and the development of specialist multidisciplinary services. However, there is substantial variation in the data collected and reported in relation to delirium and cognition, which impedes international comparison and may reduce quality of care.

**Objective:**

This scoping review aims to identify delirium and cognition data items currently collected by hip fracture registries internationally, to identify associated registry guidance that exists for the administration of delirium and cognitive screening tools, and report outcomes of these data items across the most recently published annual reports of identified hip fracture registries.

**Methods:**

This scoping review will be conducted in accordance with the Preferred Reporting Items for Systematic Reviews extension for Scoping Reviews (PRISMA-ScR). We will search the following databases: Medline Ovid; Embase; CINAHL EBSCOHost. Relevant websites such as the Fragility Fracture Network (FFN) will also be searched. Study selection and review will be carried out independently by two research team members, with discrepancies resolved by a third member of the research team. Data extraction and synthesis will be conducted by one reviewer and checked for accuracy and omissions by another. The scoping review findings will be informed and validated through engagement with the FFN Hip Fracture Audit Special Interest Group, who will share their knowledge, expertise, and research to achieve consensus over core aspects of the scoping review findings.

**Conclusion:**

By identifying existing heterogeneity in delirium and cognitive screening tool use and administration, it is hoped that administration and specific screening tool use will become standardised to optimise comparability across countries and ensure that high quality and reliable data are included across international registry reports.

## Introduction

Hip fracture is one of the most serious consequence of falls among adults ^1^, and older adults with hip fracture are at increased risk of neurocognitive disorders (NCDs) ^2^. Delirium is an acute and fluctuating NCD, typified by altered awareness, impaired attention, and other cognitive and neuropsychiatric disturbances ^3^. Mild cognitive impairment is an NCD, characterised by a disruption to some cognitive function, such as memory, with preserved independence in functional abilities ^4, 5^. Dementia is an NCD in which there is deterioration in cognitive function beyond that which might be expected from the usual consequences of biological ageing, and which affects independence in functional ability ^6^. Systematic review evidence suggests that 19% of people with hip fracture have a pre-fracture diagnosis of dementia, 42% have cognitive impairment ^7^, and 20–53% experience delirium ^2, 8, 9^. Although delirium is the most common complication following hip fracture ^10^, there is evidence to suggest that up to one third of cases are preventable ^11^. Older adults with cognitive impairment or dementia are more likely to experience delirium and to have a complex hip fracture care pathway, due to their greater risk of poor outcomes and need for tailored support ^12^. Delirium can result in a higher rate of discharge to long-term care, higher mortality, reduced mobility, and increased care needs ^13, 14^. Awareness of baseline cognitive function and assessment of cognitive function are essential facets of clinical assessment in people with hip fracture and can significantly impact on decisions regarding type of surgical management, rehabilitation, and discharge planning ^15, 16^, as well as physical function in the first year following hip fracture surgery ^17^.

Founded in 2011, the Fragility Fracture Network (FFN) is a global multidisciplinary organisation that aims to improve the management and secondary prevention of fragility fractures while also promoting the wider establishment of hip fracture registries. National hip fracture registries facilitate improvements in outcomes and care quality, such as timely surgery, early post-operative mobilisation, reduced mortality, and the development of specialist multidisciplinary services ^18– 21^. A review of European national hip fracture registries found that hip fracture registries were an effective tool for comparison of hospitals within one country, however the presentation and collection of data need to become more uniform to facilitate international comparisons ^22^. To improve comparability across national registries and to assist in the development of new registries, a minimum common dataset (MCD) was initially introduced in 2014 and reviewed in 2022 by the FFN Hip Fracture Audit Special Interest Group, which comprises nominated representatives from each of the established national hip fracture programmes, international FFN representatives, and senior figures from the FFN administration ^23, 24^. The MCD represents the simplest dataset that new national audits should aim to collect. It includes 22 core questions and 12 optional fields, which may be used depending on the health system structure and organisation of different countries ^24^.

Assessment of cognitive status is one of the core questions of the MCD. However, the use of a specific tool, timing, and frequency were not recommended due to barriers such as language, local validation of tools, and cultural differences across countries. Variations in definitions and tool use can thus be challenging when attempting to compile and compare data across registries and so registries are advised to provide accurate data dictionaries ^23^. A 2023 review of comparability of hip fracture registries based on their use of the MCD highlighted the assessment of pre-fracture cognition on admission as one area where interoperability across registries could be improved ^16^, with only 65% of included registries assessing cognitive status. Similarly, the method of cognitive assessment varied across the registries collecting these data, ranging from the use of a standardised tool to the basic recording of any suggested history of dementia.

Identifying people with hip fracture who experience delirium has become a care quality imperative ^16^. Several national guidelines and care standards urge routine delirium screening, which is an essential facet of acute geriatric care ^25– 29^. However, there is no international consensus on assessment methods or a recommended tool. Some national hip fracture registries, such as the Scottish Hip Fracture Audit and the Spanish National Hip Fracture Registry (RNFC), have now included screening for delirium as part of their standards for hip fracture care ^25, 30^, and recommend use of the 4AT rapid clinical test for delirium ^30, 31^. The impact of impaired cognition and pre-operative delirium on hip fracture outcomes requires further substantial investigation, which could be expediated with increased consistency of definitions and assessment tools across hip fracture registries ^16, 32^. It is therefore timely to conduct a scoping review of data items relating to delirium and cognitive screening to build on the work of previous reviews and improve consistency in data collected by hip fracture registries.

The aim of this scoping review is to identify delirium and cognition data items that are currently collected by hip fracture registries internationally, to identify guidance that exists for the administration of delirium and cognitive screening tools across hip fracture registries, and to report outcomes of these data items across the most recently published annual reports of identified hip fracture registries.

## Methods

This scoping review will follow the Joanna Briggs Institute (JBI) methodology for conducting scoping reviews ^33, 34^ and subsequent JBI guidance for engagement with knowledge users ^35^. JBI methodology for scoping reviews includes the following stages: 1) Defining and aligning the objective(s) and question(s); 2) Developing and aligning the inclusion criteria with the objective(s) and question(s); 3) Describing the planned approach to evidence searching, selection, data extraction, and presentation of the evidence; 4) Searching for the evidence; 5) Selecting the evidence; 6) Extracting the evidence; 7) Analysis of the evidence; 8) Presentation of the results; and 9) Summarising the evidence in relation to the purpose of the review, making conclusions, and noting any implications of the findings. The scoping review findings will be reported in accordance with the Preferred Reporting Items for Systematic reviews and Meta-Analysis extension for Scoping Reviews (PRISMA-ScR) reporting guidelines ^36^.

### Engagement

The review authors are all working in the field of hip fracture audit, or evidence synthesis of audit/registry data, and are invested in improving outcomes for people with hip fracture. This scoping review will include engagement with knowledge users, including Chairs of national hip fracture registries, through liaison with the Fragility Fracture Network (FFN) Hip Fracture Audit Special Interest Group. We will also engage regularly with the Irish Hip Fracture Database Governance Committee, which is a multi-stakeholder group comprising clinical leads, healthcare professionals, and patient representatives who make strategic decisions in relation to the hip fracture registry. The knowledge users will support the scoping review in the following ways:

• Knowledge users will be asked to assist in the identification of relevant hip fracture registries from their areas of knowledge and expertise.
• Knowledge users will be presented with all findings that are identified from the scoping review and will offer expertise in validating the findings.
• Knowledge users will be asked to assist in disseminating the scoping review findings to the international hip fracture care community.
• Knowledge users who have made a substantial commitment that meets the International Committee of Medical Journal Editors (ICMJE) authorship requirements, will be eligible for authorship on the final report.

### Review questions

What data items relating to delirium and cognition are currently collected by hip fracture registries internationally?

I. What guidance exists for the administration (e.g., timing, frequency) of delirium and cognitive screening tools across hip fracture registries?
II. What delirium and cognitive screening tool outcomes are reported in the most recently published annual reports of identified hip fracture registries?

### Eligibility criteria

Eligibility criteria for this scoping review will follow the PCC mnemonic (Population, Concept, and Context) for scoping reviews ^34^.

***Population.*** The target population will be people with hip fracture. The term hip fracture is used to describe a break or fracture in the upper portion of the femur where the bone meets the pelvis. Hip fracture can be referred to as a ‘broken hip’, a ‘fractured neck of femur’ or a ‘proximal femur fracture’ ^37^. Therefore, a broad definition of hip fracture will be used. Non-hip fracture populations will be excluded.

***Concept.*** The concepts of interest are national hip fracture registries that involve data collection for the purpose of monitoring and improving the quality of hip fracture care at a country level, associated registry guidance for administration of delirium and cognitive screening tools, and the most recent annual report of identified registries. National registries offer a comprehensive portrayal of everyday practice and regional variability as they aim to cover all incident hip fracture cases within a country, which is reflective of how a healthcare system functions as a whole, rather than single centres or institutions ^38^. For inclusion, a hip fracture registry will be considered as being at country level if it is reported as the accepted country-wide structure for data collection, or if it includes the country’s name or the word ‘national’ in the title. Registries will be excluded if they operate at regional or institutional levels that are not part of a national network or do not inform national performance reviews. In addition, the registry must be in operation in 2024. Continuous data collection is imperative for monitoring time trends in hip fracture epidemiology and the effects of practices implemented by healthcare professionals or health systems ^38^. Registries without continuous data collection will be excluded.

***Context.*** The review context is an international focus on hip fracture care, primarily in acute settings, though other settings will be considered if delirium or cognitive screening is documented to occur at other stages in the hip fracture care pathway.

***Evidence sources.*** Eligible hip fracture registries will be identified from peer-reviewed articles that signpost to the relevant registry, through searches of relevant websites such as the Fragility Fracture Network (FFN), and through engagement with knowledge users and the FFN Hip Fracture Audit Special Interest Group. Conference abstracts will be excluded. Once an eligible hip fracture registry is identified, the registry website will be searched for data dictionaries, guidance relating to administration of delirium and cognitive screening tools, and the registry’s most recent annual report. Non-English registry documents will be translated by a translation service company and included in the data extraction and synthesis stages.

### Relevant studies

A comprehensive search strategy will be developed and piloted against exemplar articles to ensure the sensitivity and specificity of the search strategy and to ascertain whether further refinement is necessary. A search of electronic databases will be conducted, including MEDLINE (Ovid), Embase (Elsevier), and CINAHL (EBSCO). No date or language restrictions will be applied. Relevant websites, such as the FFN and organisational websites associated with hip fracture registries identified from exemplar articles, will be searched through use of the Google search engine. Citation lists of previous reviews and included studies will be hand-searched. Chairs of national hip fracture registries, identified through the FFN Hip Fracture Audit Special Interest Group, will be consulted to assist in the identification of relevant hip fracture registries from their areas of knowledge and expertise. See Extended data for full search strategy for all sources ^39^.

### Study selection

Two reviewers will independently review the titles and abstracts of the citations identified from the search strategy to select those for full-text review. The full texts will be obtained and independently assessed by two reviewers, applying the defined inclusion and exclusion criteria. Where disagreements occur, discussions will be held to reach consensus, or an additional reviewer will be consulted. Citations excluded during the full-text review stage will be documented alongside the reasons for their exclusion and included in the PRISMA flow diagram ^40^.

### Data extraction

A standardised extraction form specifically designed for this review will be used independently by two reviewers to pilot data extraction on three registries to calibrate and test the form. Once consensus has been reached and the extraction form has been finalised, one reviewer will extract the remaining data, and a second reviewer will check for accuracy and omissions. Any disagreements between reviewers will be resolved through discussion or through consultation with a third reviewer. Where it is unclear what delirium or cognitive screening tools are used, or at what timepoint, registry/audit managers will be contacted. Data will be extracted at two levels: i) Hip fracture registry context and characteristics, ii) associated registry guidance for delirium and cognitive screening tool administration, and delirium and cognitive screening outcomes as reported by the most recent annual report of identified registries.

For the first level, where data are extracted about the hip fracture registry itself, the extraction will include details specific to the registry characteristics and context, such as setting, age of patient inclusion, years of operation, types of cases included, phases of care covered, number of sites, level of coverage, standards of care relating to delirium and/or cognition, follow-up, and whether participation is mandatory or voluntary. For data items relating to delirium and cognitive screening, the question/specific tool used and potential response options, and the applicable/eligible patient population for each item will be extracted and tabulated. The rationale for inclusion of each item will also be identified, where possible. Associated registry guidance for administration of delirium and cognitive screening tools will be extracted, where possible. Relevant guidance and rationale relating to recommended delirium or cognition tool use, timing of screening, healthcare professional responsible for screening, and frequency of screening will be extracted and tabulated. For delirium and cognitive screening outcomes reported by the most recent annual report of identified registries, data will be extracted and tabulated relating to the total number of patients included in the annual report, the total number of patients screened for delirium and/or cognitive impairment at each timepoint, where available, and the total number of patients who tested positive for delirium or cognitive impairment at each timepoint.

### Data synthesis and presentation

All identified eligible hip fracture registries will be listed and described in detail. A checklist of the 50 most populous United Nations (UN) member states or constituent countries of a UN member state will be compiled for comparison with identified hip fracture registries to illustrate their international distribution or potential gaps. Data items and associated guidance relating to delirium and cognitive screening included in each hip fracture registry will be presented in tables. Additionally, delirium and cognitive screening outcomes reported by the most recent annual report of identified registries will be presented as per data extracted. These outcomes will then undergo descriptive analysis and be reported as frequencies.

### Dissemination of findings

Findings from this scoping review will be disseminated via publication in an academic international peer reviewed journal. Findings will also be presented at national and international conferences targeting the hip fracture care community. We also aim to disseminate the findings through the Fragility Fracture Network.

## Discussion

### Conclusion

This scoping review will contribute to a larger project, entitled “The HipCog Study”, aimed at evaluating outcomes for older adults with neurocognitive disorders and hip fracture. By identifying existing heterogeneity in delirium and cognitive screening tool use and administration, it is hoped that administration and specific screening tool use will become standardised to optimise comparability across countries and ensure that high quality and reliable data are included across international registry reports.

## Ethics and dissemination

Ethical approval and written consent were not required.

## Data Availability

No data are associated with this article. Zenodo: Extended Data for ‘Delirium and Cognitive Screening in National Hip Fracture Registries: Scoping Review Protocol. https://zenodo.org/doi/10.5281/zenodo.13643122 ^39^ This project contains the following extended data: Full Search Strategy Data Extraction Template Data are available under the terms of the Creative Commons Attribution 4.0 International license (CC-BY 4.0). Formal screening of search results against the eligibility criteria is ongoing.
