# Supplementary material for: Delirium and Cognitive Screening in National Hip Fracture Registries: Scoping Review Protocol
Source: HRB Open Res. 2025 Jan 24;7:68. Originally published 2024 Oct 28. [Version 2] doi: 10.12688/hrbopenres.13996.2 (PMC11773136; doi:10.12688/hrbopenres.13996.2)
Supplement: Peer review history

# Reviewer report: Reviewer response for version 2

**DOI:** 10.21956/hrbopenres.15459.r44987

**Published:** 2025-01-27

**Authors:** Ole Martin Steihaug

**Affiliations:** 1 University of Bergen, Bergen, Norway

Thank you to the authors for considering my comments and revising the paper and the associated files at the data repository. I have no further comments and recommend that paper is indexed.

Is the study design appropriate for the research question?

Yes

Is the rationale for, and objectives of, the study clearly described?

Yes

Are sufficient details of the methods provided to allow replication by others?

Partly

Are the datasets clearly presented in a useable and accessible format?

Not applicable

Reviewer Expertise:

hip fractures, cognitive impairment

I confirm that I have read this submission and believe that I have an appropriate level of expertise to confirm that it is of an acceptable scientific standard.

# Reviewer report: Reviewer response for version 1

**DOI:** 10.21956/hrbopenres.15362.r44726

**Published:** 2025-01-22

**Authors:** Anette Hylen Ranhoff

**Affiliations:** 1 University of Bergen, Bergen, Norway

This is a protocol article describing the rationale for, the aims and the methods of a scoping review of delirium and cognitive screening in National Hip Fracture Registries.

 It is well known that cognitive impairment, dementia and delirium are very common among hip fracture patients and have great impact on care and recovery.

 So this is a priceworthy initiative. The study protocol is clear and well written, and I have only some minor comments to make:

 It is of interest to know how many countries who have national hip fracture registries. This could be included in this protocol article, or be a result from the actual review.

 It is a strength that the review aim to differentiate between delirium, mild cognitive impairment and dementia, which are conditions with different consequences who might require different screening tools. 

Is the study design appropriate for the research question?

Yes

Is the rationale for, and objectives of, the study clearly described?

Yes

Are sufficient details of the methods provided to allow replication by others?

Yes

Are the datasets clearly presented in a useable and accessible format?

Not applicable

Reviewer Expertise:

My background is specialist in internal medicine and geriatrics, now professor emerita from University of Bergen. I have many years experience as orthogeriatrician caring for older people with hip fractures and knowledge on hip fracture registries. I have been involved in the Fragility Fracture Network (FFN) as a board member and for a periode (2022-23) as chair of the Scientific Committee. I am still involved in planning og congresses and as a boardmember of FFN Norway. Except for my involvement in FFN, I have noe conflicts of interests as reviewer of this paper.

I confirm that I have read this submission and believe that I have an appropriate level of expertise to confirm that it is of an acceptable scientific standard.

# Response

**Published:** 2025-01-22

**Authors:** Niamh A. Merriman

**Affiliations:** University College Dublin, Dublin, Ireland

We thank the reviewer for their constructive feedback on this protocol. We now address each point in responses below the review comment.

*This is a protocol article describing the rationale for, the aims and the methods of a scoping review of delirium and cognitive screening in National Hip Fracture Registries.*

*It is well known that cognitive impairment, dementia and delirium are very common among hip fracture patients and have great impact on care and recovery.*

*So, this is a priceworthy initiative. The study protocol is clear and well written, and I have only some minor comments to make:*

- **Response**: Thank you for reviewing this protocol. We appreciate the feedback on relevance of the study.

*It is of interest to know how many countries who have national hip fracture registries. This could be included in this protocol article or be a result from the actual review.*

*It is a strength that the review aim to differentiate between delirium, mild cognitive impairment and dementia, which are conditions with different consequences who might require different screening tools.*

- **Response**: Thank you for this comment. We appreciate your positive feedback on our study’s aim. In terms of how many countries have national hip fracture registries, we hope to answer this question through the review process and through engagement with the FFN Hip Fracture Audit Special Interest Group. We have also added the following to the Data synthesis and presentation section: “ *A checklist of the 50 most populous United Nations (UN) member states or constituent countries of a UN member state will be compiled for comparison with identified hip fracture registries to illustrate their international distribution or potential gaps*.”

# Reviewer report: Reviewer response for version 1

**DOI:** 10.21956/hrbopenres.15362.r44483

**Published:** 2025-01-20

**Authors:** Ole Martin Steihaug

**Affiliations:** 1 University of Bergen, Bergen, Norway

This is the protocol of a scoping review of how national hip fracture registries currently assess cognitive status with a focus on delirium and dementia. The authors hypothesize that there is wide variability in how delirium and dementia is assessed, and hence that the quality of the information is poor. The authors hope that identifying heterogeneity in care, will lead to greater standardization and ease comparisons between countries.

 I have experience with hip fracture registries, cognitive impairment and scoping reviews and I am able to review this paper. I don’t have any conflicts of interest of relevance to the paper, the authors, or my job as a reviewer.

 The paper is published is and openly accessible at the time of review. I agree that the topic is relevant and that publication of the protocol before the paper is published will lead to greater trust in the findings. The paper is well written and it is easy to understand. The introduction is informative. From an international and patient perspective it is important to gauge if the typical hip fracture patient is entered into a register and receives treatment in a health care system that uses a registry.

 The protocol is brief. I appreciate the linked information at University College Dublin’s data repository, https://zenodo.org/records/13773242 (Merriman, N. A., Penfold, R. S., Brent, L., Hickey, P., Walsh, M. E., Sexton, E., Coughlan, T., MacLullich, A. M. J., Johansen, A., Ojeda-Thies, C., Hall, A. J., & Blake, C. (2024). Extended data for 'Delirium and Cognitive Screening in National Hip Fracture Registries: Scoping Review Protocol.',  which includes a 5 page document with information on the search strategies for three different databases.

 Could you please report more extensively on the specific parts of the PRISMA guideline for scoping reviews? I suggest that you fill in the form and append it to the protocol paper.

 The protocol lists only a limited number of methods for obtaining information on hip fracture registries, using systematic searches of the academic literature, searching websites and contacting the Fragility fracture network. I note that there will be no language restrictions on the search. What resources do the authors have available to assess information in languages other than English?

 Working from the assumption that having a hip fracture register is a part of the reference standard of care for patients of hip fractures, I suggest adding a check-list of the 50 most populous countries in the world with information on whether each country has a national hip fracture registry, as assessed by your search strategy. This will hopefully illustrate blind spots in either the search strategy or the international distribution of hip fracture registries.

 I wish the authors good luck on carrying out the scoping review.

 Comments related to specific parts of the paper:

**Abstract**:

 Methods:

 “Study selection and review will be carried out independently by two researchers, with discrepancies resolved by a third researcher.” Will the researchers in question also be among the authors? Please inform. 

 “The scoping review findings will be informed and validated through engagement with the FFN Hip Fracture Audit Special Interest Group.» Can you please explain this further? The meaning is not clear to me.

 Conclusion: There is a conclusion in the abstract, but not in the main body of the paper.

**Introduction:**

 The sentence “formal diagnostic criteria for dementia” is inaccurate. Pre-fracture diagnosis of dementia is the terminology used in ( *Ref-1)*

**Methods, relevant studies:**

 “A comprehensive search strategy will be developed, and a search of electronic databases will be conducted, including MEDLINE (Ovid), Embase (Elsevier), and CINAHL (EBSCO). “ Thank you for including the full search strategy in the document at zenodo. Do you have a strategy for handling the need to refine or adjust the initial search strategy?

 “Relevant websites, such as the FFN will be searched.” Can the authors please supply additional details of what websites will be searched? Will you be accessing the websites of government health agencies? The World Health organization? Medical societies? Will you be using search engines such as google?

**Methods, study selection:**

 “The full texts will be obtained and independently assessed by two reviewers, applying the defined inclusion and exclusion criteria. « Please tell me what the exclusion criteria are.

**Methods, data extraction:**

 «A standardised extraction form specifically designed for this review will be used independently by two reviewers to pilot data extraction on three registries to calibrate and test the form. « Please include the form with the protocol.

**Data availability, Study status:**

 “Formal screening of search results against the eligibility criteria is ongoing.” What are the eligibility criteria? Can you please include them with the protocol?

 End of review

Is the study design appropriate for the research question?

Yes

Is the rationale for, and objectives of, the study clearly described?

Yes

Are sufficient details of the methods provided to allow replication by others?

Partly

Are the datasets clearly presented in a useable and accessible format?

Not applicable

Reviewer Expertise:

hip fractures, cognitive impairment

I confirm that I have read this submission and believe that I have an appropriate level of expertise to confirm that it is of an acceptable scientific standard, however I have significant reservations, as outlined above.

## References

[1] : Characteristics of fall-related hip fractures in community-dwelling elderly patients according to cognitive status. *Aging Clin Exp Res* .2008;20(5) : 10.1007/BF03325149 434-8 10.1007/BF03325149 19039285
